# Deploying efficient net batch normalizations (BNs) for grading diabetic retinopathy severity levels from fundus images

**DOI:** 10.1038/s41598-023-41797-9

**Published:** 2023-09-02

**Authors:** Summiya Batool, Syed Omer Gilani, Asim Waris, Khawaja Fahad Iqbal, Niaz B. Khan, M. Ijaz Khan, Sayed M. Eldin, Fuad A. Awwad

**Affiliations:** 1 National University of Sciences and Technology, Islamabad, 44000 Pakistan; 2 Mechanical Engineering Department, College of Engineering, University of Bahrain, Isa Town, 32038 Bahrain; 3 Depaetment of Mechanical Engineering, Lebanese American University, Kraytem, Beirut, 1102-2801 Lebanon; 4 Department of Mathematics and Statistics, Riphah International University I-14, Islamabad, 44000 Pakistan; 5 Department of Mechanics and Engineering Science, Peking University, Beijing, 100871 China; 6 Faculty of Engineering, Center of Research, Future University in Egypt, New Cairo, 11835 Egypt; 7 Department of Quantitative Analysis, College of Business Administration, King Saud University, P.O. Box 71115, 11587 Riyadh, Saudi Arabia

**Keywords:** Health care, Energy science and technology

## Abstract

Diabetic retinopathy (DR) is one of the main causes of blindness in people around the world. Early diagnosis and treatment of DR can be accomplished by organizing large regular screening programs. Still, it is difficult to spot diabetic retinopathy timely because the situation might not indicate signs in the primary stages of the disease. Due to a drastic increase in diabetic patients, there is an urgent need for efficient diabetic retinopathy detecting systems. Auto-encoders, sparse coding, and limited Boltzmann machines were used as a few past deep learning (DL) techniques and features for the classification of DR. Convolutional Neural Networks (CNN) have been identified as a promising solution for detecting and classifying DR. We employ the deep learning capabilities of efficient net batch normalization (BNs) pre-trained models to automatically acquire discriminative features from fundus images. However, we successfully achieved F1 scores above 80% on all efficient net BNs in the EYE-PACS dataset (calculated F1 score for DeepDRiD another dataset) and the results are better than previous studies. In this paper, we improved the accuracy and F1 score of the efficient net BNs pre-trained models on the EYE-PACS dataset by applying a Gaussian Smooth filter and data augmentation transforms. Using our proposed technique, we have achieved F1 scores of 84% and 87% for EYE-PACS and DeepDRiD.

## Introduction

A leading factor in blindness, diabetic retinopathy affects diabetics worldwide. A diabetic patient’s rise in blood sugar levels can result in the rupture of a tiny blood artery inside the retina, which can then bleed there. The intensity of the disease varies from normal vision to complete vision loss^1^. According to current statistics 463 million people are suffering from diabetes all around the world and out of these patients one-third have some grade of diabetic retinopathy. According to the previous literature studies, it is obvious that nearly 160 million population is affected by this disease. A drastic inclination in the DR population up to 191 million is expected by 2030^2^. DR evolves due to the constant existence of diabetes mellitus^3^. Early-stage clinical signs of DR, such as micro-aneurysms, small hemorrhages, and mild blood vessel changes, can be difficult to detect, especially without specialized equipment and training. Sometimes early-stage DR patients may not exhibit overt visual symptoms, making it challenging to identify those who require screening or therapy. Depending on the imaging environment, patient cooperation, and camera calibration, retinal images acquired during screenings may or may not be of good quality. It could be challenging to accurately diagnose and evaluate instances with poor image quality. Early stages of DR may exhibit complex patterns of lesions that require a careful assessment by competent professionals. It could be difficult for automated systems to identify and exactly explain these patterns. Healthcare facilities may have trouble managing screenings and huge data. Ultimately, results in blindness if the prognosis is not received in time for therapy^3^. A comprehensive strategy that incorporates improvements in medical imaging technology, machine learning, data management, patient education, regulatory reforms, and better healthcare infrastructure is needed to detect disease in the early stages. To guarantee accurate and prompt DR identification, successful screening programs and various retinal visionary tests should work to address these issues. This will improve patient outcomes and enable more effective use of healthcare resources^4^.

DR is commonly classified into the following groups, normal, non-proliferative diabetic retinopathy, and proliferative diabetic retinopathy established off the growth of this disease. In non-proliferative diabetic retinopathy (NPDR) fresh retinal blood vessels don’t emerge and these blood vessels’ wall has vanished. NPDR is fragmented into mild, moderate, and severe (NPDR). New blood vessels in the retinal region obstruct blood flow to the retina during the PDR phase^4, 5^. To reduce the likelihood of developing blindness, it is essential to identify DR at the earliest possible stage. Many researchers have proposed various computer-aided intelligent identification methods in the last ten years. Identification of disease can be misjudged by the naked eye and needs careful observations and multiple attempts. The computer-aided diagnostic technique saves expense and time and is more efficient than common detection procedures.

The computer-aided diagnostic techniques are divided into three categories: Computer-Aided Detection (CAD) methods, Machine Learning (ML) techniques, and Deep Learning (DL) algorithms. Computer-Aided Detection (CAD) systems were created and used in clinical workflows in the early 2000s to assist clinicians with decision-making and illness detection. However, CAD systems have limits. One of them is that the adversaries report more false positives than the human reader, requiring extra investigation, money, and time for the doctor to make a decision on the patient’s health state. Structured data is required for machine learning. Machine Learning examples include decision trees, support vector machines, linear regression, logistic regression, and), Nave Bayes, the Hidden Markov model, and Bayesian networks (statistical classifiers)^6, 7^.

Deep Learning is a machine learning subfield inspired by both the operation and structure of the brain. Deep Learning techniques may be utilized when there is sufficient computer power, enough storage capacity, and a large amount of data. Convolutional Neural Networks, a Restricted Boltzmann Machine, a Long Short Term Memory, or Sparse Auto Encoders are included in deep learning. Sparse Auto Encoders imposed sparsity in learned representations, which can aid in capturing essential retinal image properties.

Restricted Boltzmann Machines are generative models that have been utilized for feature extraction and pre-training in some circumstances. Using limited Boltzmann machines, specialized retinal pictures were analyzed and categorized. In order to identify retinopathy and normalcy patterns following the system training phase, our model retrieved 500 attributes from the pictures for disease categorization^8^. One kind of Auto Encoder that tends to force the extraction of sparse features from raw data is the Sparse Auto Encoder. Either penalizing hidden unit biases or directly penalizing the output of hidden unit activations can be used to accomplish the representation’s sparsity. Due to its reliability in recovering damaged input and compelling the model to record the right version, Denoising Auto Encoders (DAEs) have also been utilized in DR detection^9^. CNN is a discriminative, supervised model that is trained using backpropagation and the gradient descent method. Grid topology is ideal for CNN. The grid format input, such as an image (2D), is supplied through layers, which retain the spatial connection between the layers. CNN’s core building blocks are the ConvNet layers, activation layers, pooling layers, and fully linked layers. Many current CNNs contain dropout regularization and batch/group normalization. According to a review of the literature, there are several CNN architectures suggested by various researchers. AlexNet, VGG, GoogleNet, Inception, ResNet, efficient net and others are among them. An effective scaling approach for increasing the depth, breadth, and resolution of the images used in the construction of the Efficient Net, as opposed to other scaling methods that arbitrarily scale depth, height, and width. In the literature, the best accuracy and F1 score were observed by efficient net BNs rather than ResNet and VGG models^10^.

We classify DR phases using straightforward, lightweight CNN-based models. These models are not too complex and time-consuming^5^. For the diabetic retinopathy-restricted strength of training data images and variable annotations, computer-aided diagnosis systems have recently encountered two additional challenges^11^. An Efficient neural network pre-trained model has been implemented in a MONAI (PyTorch-based), deep learning open-source framework in healthcare imaging. In this study, we proved EfficientNet BNs pre-trained models to classify all five stages of diabetic retinopathy^12^. We resized it with a high resolution rather than the previous study^13^ to achieve high scores. As the model can differentiate each type of diabetic retinopathy, it may be utilized in healthcare to assist clinicians in making decisions on the patient’s model.

The rest of the paper layout consists of the following sections: The related work section is a survey of scholarly sources on a topic. It provides an overview of current knowledge, allowing to identification of relevant theories, methods, and gaps in the existing research. The methodology section outlines what we did and how we did it, allowing readers to assess the trustworthiness and validity of our study. The results section should provide findings objectively, with just brief observations in regard to each question, hypothesis, or topic. In the discussion section, the results’ significance and relevance are explained. It should concentrate on describing and analyzing what was discovered and demonstrating how it connects to the paper. In last concluded research study summarizes the research’s principal results, importance, and consequences.

## Related works

In accordance with Li et al.^1^ proposed work, methods using fundus pictures and the VGG-Net were combined to enhance classification performance. The effective preprocessed techniques used were non-local mean denoising for improved retinal picture viewing, weighted Gaussian blur, and interpolation scaling of images. The VGG Net model grade five classes and an additional grade (class 5) were applied by da Rocha et al.^3^ to reveal the poor quality of digitized retinal images that are frequently found online and available in the DDR, EyePACS, and IDRiD databases. Saranya et al.^5^ deployed the CNN layers were assessed on two online available databases and these are MESSIDOR and IDRiD. They classified four classes (0, 1, 2, 3) of diabetic retinopathy and they did not use PDR (4) stage. Preprocessing consisted of Canny Edge detection, scaling, interpolation, and normalization standardization, followed by the application of a CNN classifier to visualize the severity degree of DR. In Sai Venkatesh Chilukoti et al.^13^ paper deployed ResNet, VGG, and efficient net BNs (0–6) models. They got results evaluation using the F1 and quadratic weighted kappa scores, which were suitable for grading various classes built on the intensity. But they got the best F1 and Quadratic weighted kappa scores on efficient net B3. In their paper, ResNet and VGG models evaluate least F1 and Quadratic weighted kappa = 0 scores. Our proposed work on efficient net BNs (0–6) on two datasets evaluated the F1 score. We had improved the F1 score in all efficient net BNs but only efficient net b-3 has the same score in the EYE-PACS dataset as in previous work. For the first time evaluated the F1 score by applying efficient net BNs on DeepDRiD. Gao Jinfeng et al.^14^ two deep CNN models used an ensemble method to detect all the DR stages by using balanced and unbalanced datasets. The outcome demonstrated that models outperform more sophisticated techniques like the Kaggle datasets in contrast to how well they now detect all stages of DR. By implementing a light-weight mobile network and assessing the effectiveness of their classifier, Sheikh et al.^15^ employed a novel approach. MobileNetV2 was constructed as a light-weight, mobile-friendly architecture and trained using datasets of diabetic retinal fundus images. Yi et al.^16^ applied RA-efficient net for 2-grade and 5-grade classifications. They used the APTOS 2019 dataset. In their model RA block best to perceive between the lesion features of DR images. A new method for binary class and multiclass classification based on the datasets of APTOS (2019) blindness detection and Messidor-II was announced by Nahiduzzaman et al.^17^. Initially, data were preprocessed by applying Ben Graham’s technique. To locate the contrast-enhanced image data with the least amount of noise, contrast-limited adaptive histogram equalization (CLAHE) has been used. After that, a new hybrid CNN model singular value decomposition is proved to be useful for decreasing the input classifier. To reduce the training time, an ELM technique was employed as the classifier. Their approach focused on accuracy, precision, recall, and F1 score, showing the achievable potential of a future DR detection strategy. Khan et al.^18^ applied depth learning-based ensemble techniques for diabetic retinopathy identification. They conducted structural modifications in real CNN to increase the effectiveness and precision of grading the DR’s classes in fundus colorful images. They worked on an imbalanced Kaggle dataset to check the working of their deployed model. The results showed that the applied model did not have high accuracy computational scores^19, 20^. Recently, there have been several notable research studies in the field of biomedical applications. One study by Zeng et al.^22^ explored the use of the metal–organic framework to advance the signal of hyperpolarized xenon nuclear magnetic resonance. Another study by Ye et al.^23^ analyzed the effectiveness of vitrectomy in treating highly myopic macular holes. Ao et al.^24^ focused on stimulated Raman scattering microscopy, while Wang et al.^25^ discussed a simple low-light image enhancement technique based on the Weber–Fechner law. Wang et al.^26^ investigated an injectable polyzwitterionic lubricant for preventing cardiac adhesion.

There were also several research studies in the field of medical imaging. Zhang et al.^27^ and Liu et al.^28^ developed algorithms for endoscope imaging and analyzed applications of large CT image sequences. In the other recent research, Zhuang et al.^29, 30^ improved the image quality assessment in large lung CT images. Other studies, represented by references^31–33^, focused on different aspects of image quality assessment.

In addition to imaging, there were studies on biomedical signal processing and clinical evaluation^34–36^. Dual microphone active noise cancellation was explored in a study^37^, while other studies delved into fatigue feature detection method based on multifractal theory, 3D scanning and 3D object detection network^38–40^.

## Materials and methods

**Figure 1:**
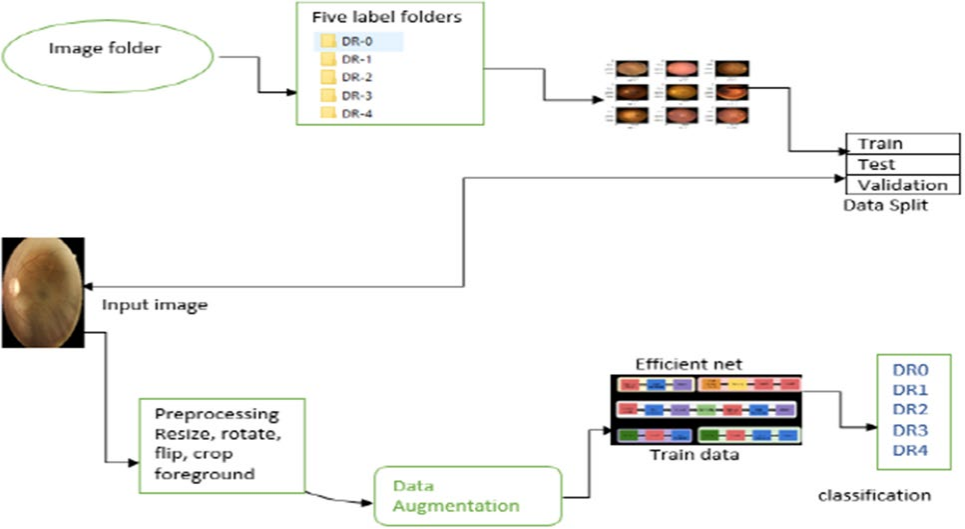
Our framework with all phases.

Our proposed framework includes the following phases: images into separate labels folders, data loading splitting train, validation and test data, preprocessing, data augmentation, and classification (as shown in Fig. 1).

### Description of datasets

**Figure 2:**
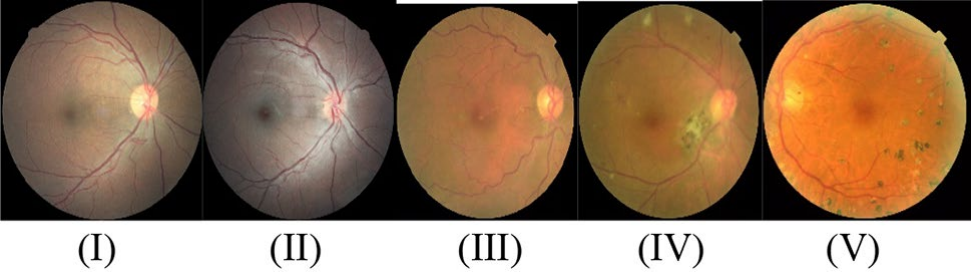
Fundus images of (**I**) no-DR, (**II**) mild-DR, (**III**) moderate-DR, (**IV**) severe-DR, (**V**) PDR.

With the help of retinal fundus images, our work presents a predicate technique for grading DR. DeepDRiD and Kaggle EYE-PACS datasets, both of which are freely accessible online, are used. In these two datasets originally retinal images were collected from subjects under the supervision of various medical institutions. These retinal images are both the left and right eyes of each subject (participant). The Ethics Committee at Shanghai Sixth People’s Hospital accepted the DeepDRiD dataset, and the study followed the Declaration of Helsinki. Participants provided informed consent. Under the identifier ChiCTR2000031184, the trial was entered into the Chinese Clinical Trials Registry (ChiCTR.org.cn)^41^. The EYE-PACS dataset images were taken at several primary care facilities in California and elsewhere using a variety of different equipment and under a variety of different circumstances. So, this is a heterogeneous dataset made up of images from various smaller datasets that were taken with various cameras, using various settings, at various sizes, and under various lighting and brightness conditions. According to the Early Treatment Diabetic Retinopathy Study (ETDRS) scale, a physician was asked to assign a score of 0–4 to each image based on the presence of diabetic retinopathy (DR)^2^. This dataset is openly available in a zip file on the Kaggle site^42^. The total labeled fundus images in DeepDRiD and Kaggle datasets are 1600 and 35,108. Compared to Kaggle EYE-PACS, and DeepDRiD have a limited database. These two databases each feature five DR phases, which are represented by the numbers 0, 1, 2, 3, and 4: normal, mild, moderate, severe, and proliferative. Figure 2 displays fundus pictures of these five stages.

These five stages are classified on the base of lesions. That is shown in the below Table 1.

**Table 1:** DR-Stages on the base of lesions.

Stages	Findings
No DR	No lesions or spots
Mild DR	Multiple hemorrhages and cotton-wool spots
Moderate DR	Presence of micro-aneurysms, intra-retinal hemorrhages, or venous beading
Severe DR	Affecting small blood vessels in the eye due to blockage or leakage
PDR	Retina starts growing new blood vessels

All image data is distributed into 5 different folders. Each folder has single-label graded images. The data distribution is displayed in the below Table 2.

**Table 2:** Data distribution.

Labels	EYE PACS	DeepDRiD
No DR	25,802	714
Mild DR	2438	186
Moderate DR	5288	326
Severe DR	872	282
PDR	708	92
Total	35,108	1600

### Separation into folders

DeepDRiD has combined images of all grades into two folders. The first training folder has 300 patients’ sub-folders and the second validation folder of 100 patients’ sub-folders. With these folders two labeled CSV (comma-separated values) files are present. In each sub-folder, there are four eye images (2 left and 2 right). And Kaggle dataset has also one folder and a single CSV file. The folder has 35,108 fundus images. We have used code to divide single image folder data into 5 separated label folders with the help of a given CSV file. We can distribute all fundus images into label folders in a very short time through code otherwise manually it will take too much time without knowing whether distribution into the following folders is correct or not.

### Data loading

**Figure 3:**
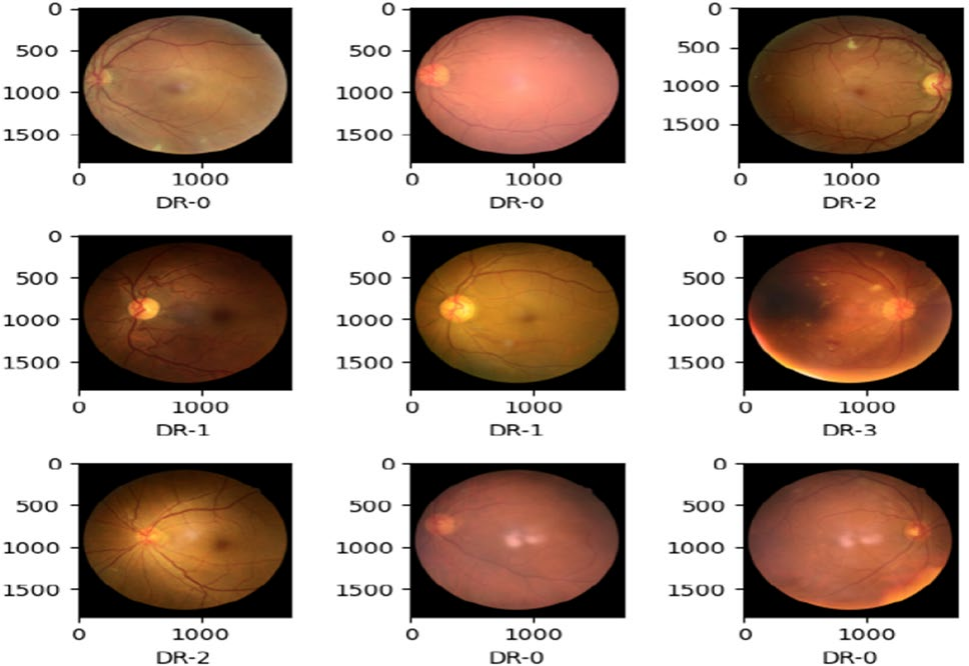
Data loader.

The data is set in the directory. The folders’ paths are given in the directory. Firstly, read the dataset files and display some statistics. To train the classification model, the dataset’s five folders normal (DR-0), mild DR (DR-1), moderate DR (DR-2), severe DR (DR-3), and PDR (DR-4) should be tagged. Images from the dataset that were viewable were randomly selected. Create a dataloader of training, testing, and validation datasets which can be seen in the below Fig. 3.

### Preprocessing

Preprocessing is the procedure that we apply before utilizing the data images for neural network training. In preprocessing we resize all image data in the same size, assess the distribution among the classes, and examine the visual quality of all classes. The preprocessing transforms are defined before adding them to data loaders. Compose Transform is used to order a series of callables collected in a sequence. Every transformation in the series essentially takes only an argument and back to one value. In our proposed model we use simple and few preprocessing techniques. In preprocessing, we applied the following transformations. These are mentioned below:

#### Crop foreground

We have applied the Crop Foreground transform to manipulate images. We have isolated the foreground and all black pixels everywhere else. After it, we are enabled to crop the image so there are no full black rows above and below, or full black columns left and right of the foreground.

#### Resize

We are using two different types of datasets, and both have different numbers of fundus images. These datasets have contrary image resolutions. In the Kaggle EYE-PACS dataset, the images have (1024, 1024) resolution. It is a huge dataset that’s why for speeding up the training we resized the images’ (512, 512) resolutions. And in DeepDRiD images have (1956 × 1934) resolution we resized the images to (512, 512) resolution. We haven’t used too many complex preprocessing methods. And resizing image data is the main transform. After resizing images, we observed speedy and prominent changes in training.

#### Gaussian smooth

There is a noise in image data. We have overcome the noise by applying a filter. During preprocessing of the data, we used the Gaussian Smooth filter. Gaussian Smooth Filter is a low pass filter applied for lowering the noise (high freq. components and distorting areas of data images). It is applied as an Odd sized Symmetric Kernel which is distributed over individual pixels of the ROI-Region of interest to develop the wanted effect. Gaussian filters are commonly isotropic, that is, they have exactly similar standard deviations along both dimensions. So, this filter removes extra noise from image data. After using this filter, we observed a prominent effect on evaluation scores.

### Data augmentation

DL mostly tracks a problem where data images have a specific size. To obtain improved generalization in the model we require more data and so as many changes as likely in the images. Occasionally dataset is not sufficient to detect adequate variation, in such scenarios, we need to create additional data from a certain dataset. In such cases, augmented data can play a significant role.

This technique is used to artificially grow the size of the training set by generating modified data from the initial one. Data augmentation transforms are applied on each grade to avoid data misbalancing. By this technique, we can prevent overfitting, and good for enhancing the model’s performance and decreasing the number of false positives.

The transforms used in data augmentation are mentioned below:

- RandRotate (range_x = np.pi/12, prob = 0.50, keep size = True);
- RandFlip (spatial axis = 0.0, prob = 0.50);
- RandZoom (min zoom = 0.90, max zoom = 1.1, prob = 0.50);
- Rand Gaussian Sharpen (prob = 0.5).

Above all, these image augmentation methods have made the model more long-lasting and generalizable to poor-quality images.

### Network structure

**Figure 4:**
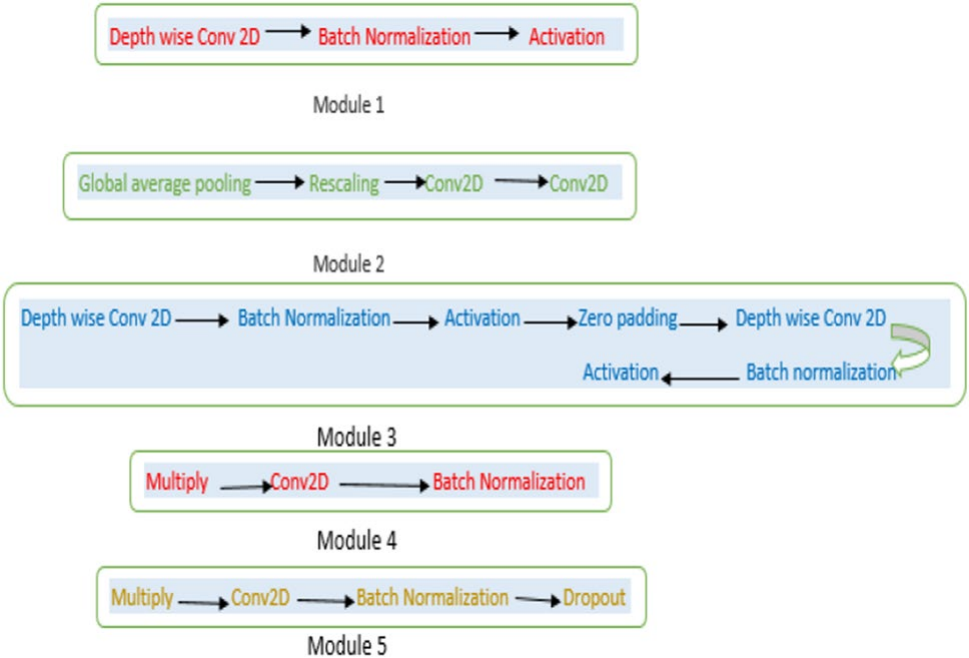
The architecture of the efficient net model.

There are numerous CNN models being used to classify the DR classes. efficient net appears to have performed admirably. This is because, in contrast to scaling techniques that arbitrarily scale depth, height, and breadth, a constructive scaling approach is used to increase the resolution, depth, and width of the images used in the architecture of the efficient net. efficient net is one of them which gives high accuracy scores with fewer parameters than other models that have high parameters. In the past Mingxing Tan and Quoc V. Le of Google Research, the Brain team introduced the efficient net categorization model. In order to create an autonomous network of neural networks that mutually optimizes accuracy and efficacy measured in FLOPS (floating point operations per second) they first created a network by carrying out a neural architecture search. This classification model’s design customs the mobile inverted bottleneck convolution (MBConv). After that, researchers expanded the fundamental network to create the efficient nets deep-learning classifiers. Before model scaling was not changed layer operators in the baseline network, having a good baseline network is similarly serious. In addition to learning a new mobile size baseline called efficient net, we are computing such a scaling approach using real Conv-Nets, which better reflect the competency of the scaling strategy. The effectiveness of network scalability will be greatly influenced by the initial network used. In order to follow the AutoML MNAS framework, which aims to compute accuracy and efficiency, the architecture used as the baseline model on efficient net is called EfficientNet-b0 (FLOPS). The basic network is then built up using the compound scaling principle to create the family of Efficient Nets, which includes efficient net b1 through EfficientNet-b7. The Efficient Net-b0 algorithm used in this study requires a mini-input size of 224 × 224 before the image size in the RMFD is smaller than 200 × 200 pixels^21^. Efficient net (BNs) models’ architecture is mentioned below in the flow diagram in Fig. 4.

Efficient net (BNs) model parameters and flops are mentioned which have been used to train our datasets in Table 3.

**Table 3:** Parameters and flops of efficient net BNs.

Model	Parameters (M)	Flops (B)
Efficient net b0	5.3	0.39
Efficient net b1	7.8	0.70
Efficient net b2	9.2	1.0
Efficient net b3	12	1.8
Efficient net b4	19	4.2
Efficient net b5	30	9.9
Efficient net b6	43	19

These are descriptions of parameters of model batch normalizations from 0 to 6. In the previous study, only an efficient net b3 gave the highest score. In their studies, they describe efficient net b3 architecture in the proposed work. According to them the efficient net b3 to develop a feature with dimensions of 7 × 7 × 1536, then a feature vector having dimension 1536 has been fully connected layer, giving a feature vector of size 512. Then passes to the dropout layer with a rate equal to 0.5, followed by the rectified linear unit (ReLU) activation layer distributed to the fully connected layer, giving the output size of 512, which is served to the dropout layer with a rate equal to 0.25 and next going to the ReLU activation layer. In the last, the output of the ReLU activation layer is given to the fully connected layer, which has five units equal to the number of output classes. We also deployed the same pre-trained efficient net BNs models as previously^13^. The only difference between their and our work is having different approaches in preprocessing and data augmentation techniques through which our all efficient net b0 to b6 models showed above 80% scores in all metrics which is state-of-the-art work.

Efficient net BNs models are renowned for their compact size. With fewer parameters than popular deep learning architectures like Res-Net, inception, and VGG, they deliver performance that is competitive. Efficient net models use less memory during training and inference since they contain fewer parameters. Efficient net models frequently exhibit lower FLOPs while maintaining similar or greater performance when compared to alternative deep learning architectures. Efficient net models are more suited for real-time applications and deployment on devices with limited resources because of the decreased computational complexity. Due to its computational effectiveness, efficient net models are suitable for deployment on devices with limited resources, such as mobile devices. Efficient net models offer a fair balance of accuracy and computational effectiveness. They usually outperform earlier designs in terms of accuracy while using less computational power than larger state-of-the-art models. Based on computational capabilities and work requirements, several model sizes (b0 to b6) are available from the efficient net family. Practitioners may make informed decisions based on their unique demands thanks to the scalability of the solution.

### Training

Efficient net b (0–6) was used to train our datasets through the use of hyper parameters. Adam’s optimizers were used to implement training for the displayed model, and the learning rate was set to 0.0001. We divided the EYE-PACS data into three groups for training, testing, and validation. 24,576 fundus images make up the training set, while 5266 images were included in the test and validation datasets. In data of DeepDRiD 1408 retinal fundus images in training data, in contrast, the test and validation dataset has 96 images. For EYEPACS and DeepDRiD, the weights of the network were randomly initialized with batch sizes ranging from 30 to 12. Then, for the EYE-PACS dataset, we trained a network for 30 epochs, and for the DeepDRiD dataset, for 100 epochs. An objective function was the categorical cross-entropy. These hyper-parameters are given below in Table 4.

**Table 4:** Hyper-parameters in training.

Serial no.	Hyper-parameters	Values
1	Learning rate (LR)	0.0001
2	Epochs (EYE-PACS)	30
3	Epochs (DeepDRiD)	100
4	Optimizer	Adam
5	Loss function	Categorical cross-entropy

## Results

By deploying efficient net BNs, a complete survey of the functioning of the proposed strategy to calculate DR. The EyePACS and DeepDRiD datasets were later applied with augmentation techniques divided into the testing and training datasets.

### The system configurations

Efficient net BNs models have been implemented in MONAI open-source architecture. The output channels of models are analogous to five classes (DR0, DR_1, DR_2, DR_3, and DR_4). We used Cross Entropy Loss as a loss function. A system with some parameters was utilized for the methodology.

- Python 3.8.8.
- Ubuntu Version 20.04.4 LTS.
- NVIDIA Tesla T4 GPU with 16 GB memory.
- RAM 32 GB memory.
- CUDA version 11.4.
- Deep learning architecture Pytorch 1.9.0 and MONAI 0.8.

### The performance metrics

We employed common and well-known metrics to evaluate the performance of our suggested technique, such as accuracy (Acc), precision, recall, and F1-score. The terms used here are used to describe how metrics are calculated.

True-Positive (TP) indicates a retinopathy class that was correctly anticipated.

The projected label that is incorrectly predicted as a retinopathy class is shown as False-Positive (FP).

True-Negative (TN) is the projected label that is really a non-retinopathy pixel False-Negative (FN) is the projected label that has been mislabeled as a non-retinopathy pixel. All metrics used in evaluating the scores are described below with formulas^9, 11, 21^.

#### F1 score

It is helpful to calculate the harmonic average of recall and precision as:

1 $$\text{F1 score} =\frac{ 2 \times precision \times recall}{precision+ recall}.$$

#### Precision

Specifically, the ratio of true positives to components is stated as being acceptable for the positive class (i.e., the sum of true positives (TP) and false positives (FP)). To show the precision, the Positive Predictive Value (PPV) is used. An equation for precision is:

2 $$\text{Precision }=\frac{TP}{TP+FP}.$$

#### Recall

It can be expressed as the proportion of TN parts to all parts belonging to the negative class (such as the total of FP and TN). The mathematical representation of the appearance is:

3 $$\text{Recall }= \frac{TN}{TN+FP}.$$

#### AUC

It is the area under the curve and has been used to execute a fixed integral among the two points. The evaluating equation is:

4 $$\text{Area under the curve}=\frac{1}{2}\left(\frac{\text{True positive}}{\text{True positive}+\text{False negative}}+ \frac{True\, negative}{True\, negative+False\, positive}\right).$$

#### Accuracy (Acc)

It indicates the percentage of correct predictions. An equation for accuracy is:

5 $${\text{Acc}} = \frac{{{\text{TP}} + {\text{TN}}}}{{{\text{TP}} + {\text{TN}} + {\text{FP}} + {\text{FN}}}}.$$

### Result evaluation

F1 scores were calculated by using six efficient net BNs classification models. The efficient net models could distinguish all classes. In addition, the efficient net BNs have identified all the classes with great possibility; later results evaluated in four metrics. We discussed three tables evaluating the scores of precision, recall, accuracy (Acc), and F1-score metrics for all the efficient net BNs models given below. Table 5 results of these four metrics of previous work^13^, on which we proposed our work. They also used the EYE-PACS dataset to evaluate these metrics.

**Table 5:** Evaluating metric scores for all the efficient net BNs of previous work.

Model	Precision	Recall	Acc	F1-score
Efficient net b0^14^	0.69	0.77	0.77	0.70
Efficient net b1^14^	0.69	0.78	0.78	0.72
Efficient net b2^14^	0.67	0.77	0.77	0.71
Efficient net b3^14^	0.83	0.85	0.85	0.84
Efficient net b4^14^	0.69	0.77	0.77	0.70
Efficient net b5^14^	0.52	0.72	0.72	0.72
Efficient net b6^14^	0.70	0.77	0.77	0.72

In the below table, we discussed these four metrics of our work on the EYE-PACS dataset.

We also evaluated metrics on another dataset (DeepDRiD) and we see prominent improvements in results in the below Table 7. We downloaded this dataset from the DeepDRiD online challenge of 2020.

We have compared the previous^13^ and our proposed work results of the F1 score metric and added execution time for each image in the below Table 8.

In the above Table 5. metrics scores of previous work utilized a Kaggle EYE-PACS dataset and deployed the model of efficient net b3, which was capable of detecting stages of DR with an achievement of F1 score of 0.84^13^. And Table 6 displayed our proposed work using EYE-PACS datasets and established all models efficient net b(0–6), which are accomplished to detect all DR stages F1 scores above 80% while only an efficient net b3 had achieved 84% F1 score in previous work with similar dataset. However, we deployed these models on DeepDRiD and evaluated the F1-score for the first time displayed in Table 7. In comparison Table 8 we observed our proposed studies displayed promising improvements in efficient net b0, b1, b2, b4, b5, and b6 in EYE-PACS. Even there were better metrics scores in the DeepDRiD dataset which evaluated the F1-score very first time with the deployment of these models.

**Table 6:** Evaluating metric scores for all the efficient net BNs on EYE-PACS.

Model	Precision	Recall	Acc	F1-score
Efficient net b0	0.81	0.83	0.83	0.82
Efficient net b1	0.83	0.85	0.85	0.83
Efficient net b2	0.84	0.83	0.83	0.84
Efficient net b3	0.84	0.85	0.85	0.84
Efficient net b4	0.83	0.84	0.84	0.83
Efficient net b5	0.83	0.84	0.84	0.83
Efficient net b6	0.83	0.85	0.85	0.84

**Table 7:** Evaluating metric scores for all the efficient net BNs of deep DRiD.

Models	Precision	Recall	Acc	F1-score
Efficient net b0	0.80	0.79	0.79	0.78
Efficient net b1	0.87	0.84	0.84	0.85
Efficient net b2	0.84	0.84	0.84	0.84
Efficient net b3	0.83	0.81	0.81	0.81
Efficient net b4	0.84	0.83	0.83	0.83
Efficient net b5	0.87	0.88	0.88	0.87
Efficient net b6	0.87	0.86	0.86	0.86

**Table 8:** Comparison of F1 scores of previous and our work.

Models	Previous work (EYE-PACS) F1-score	Our work (EYE-PACS) F1-score	Our work (DeepDRiD) F1-score	Execution time per image (min)
Efficient net b0	0.70	0.82	0.78	0.001
Efficient net b1	0.72	0.83	0.85	0.001
Efficient net 2	0.71	0.84	0.84	0.012
Efficient net 3	0.84	0.84	0.81	0.015
Efficient net 4	0.70	0.83	0.83	0.011
Efficient net 5	0.72	0.83	0.87	0.01
Efficient net 6	0.72	0.84	0.86	0.02

**Figure 5:**
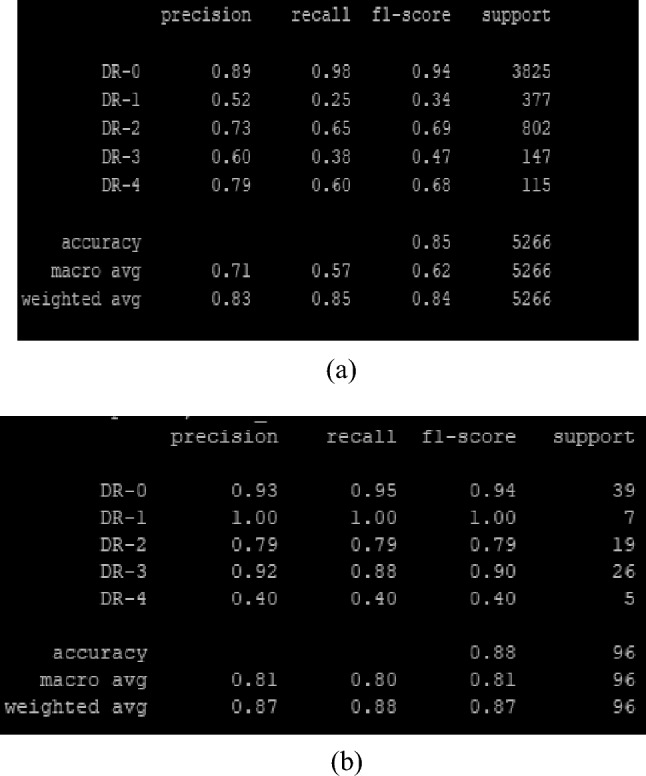
(**a**) EYE-PACS dataset (efficient net b6). (**b**) DeepDRiD dataset (efficient net b5).

The highest F1 scores of all classes of DR in both datasets are shown below in Fig. 5a,b.

**Figure 6:**
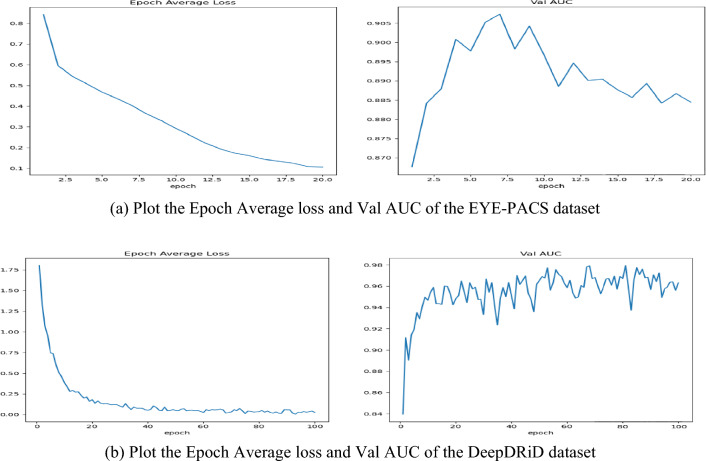
(**a**) Plot the epoch average loss and Val AUC of the EYE-PACS dataset. (**b**) Plot the epoch average loss and Val AUC of the DeepDRiD dataset.

The projected model displayed greater accuracy for identifying and sorting diabetic retinopathy on DeepDRiD and Kaggle EYE-PACS datasets. In Fig. 6a,b, epoch average and validation AUC graphs of DeepDRiD and EYE-PACS are drawn.

## Discussion

Scientists are noticing broad investigations to describe DR classes. There is a significant aim for selecting this disease in the research that this disease is frequent in China, the USA, and India, and has in recent times taken place to develop generally in our country. In future research, we will work on planning an effective DR classification system in which numerous models can be cohesive with a web-based interface for usage in clinical purposes.

In order to retain high accuracy while balancing model size and computational performance, efficient net BNs were particularly created. As a result, compared to other models, it may attain comparable performance with fewer parameters. The architectures of other deep learning models, such as VGG, ResNet, and Inception, differ and may not be as well-suited for effective computing. The basic drive of using these classification models is to detect all stages of diabetic retinopathy. Because efficient net BNs are built to reduce computational complexity, training, and inference processes go more quickly than other deep learning models. Real-time applications and situations with limited resources particularly benefit from this efficiency. Previously VGG and Resnets classification models have the highest probability of detecting DR-0 than others. But efficient net models detect high probability in other classes as well. An efficient net BNs model is the most effective CNN multi-classification of Diabetic Retinopathy, rather than VGGs and Resents. The focal objective of our work was to deploy such an effectual model which classifies all stages of DR with fewer parameters^13^.

Although efficient net models are intended to be flexible and effective image classifiers and are not specifically developed to diagnose certain stages of diabetic retinopathy (DR), their traits may help them identify different stages of DR. There are some distinctive characteristics of efficient net models that can aid with DR diagnosis at various stages.

(1) These models are made to capture properties at various levels of abstraction in an adaptable manner. (2) Efficient net’s compound scaling optimizes the model’s depth, breadth, and resolution. This optimization makes sure that the model is able to catch the small features that are present in photos, which are crucial for spotting early DR.

Applying a few simple preprocessing techniques, dataset resizing with high-resolution images, and a Gaussian smooth filter. We implemented efficient net models to enhance performance and categories at every stage. While we were able to achieve above 80% scores by applying efficient net (1–6) BNs models, previous work using efficient net BNs models deployed on the EYE-PACS dataset only acquired a 0.84 F1-score by efficient net B3. Our models raised the EYE-PACS Kaggle dataset’s F1 score, which was employed in earlier research^13^, but the DeepDRiD 2020 dataset, which we used, had a higher F1 score than the Kaggle dataset. In the EYE-PACS dataset, all of our classes have scores; however, in DeepDRiD, the majority of our classes have scores above 78% and one of our illness classes has been detected with a score of 100%. A few researchers had also previously worked with limited data on diabetic retinopathy. We were unaware that employing efficient net models with DeepDRiD little data did not evaluate F1 scores in the literature. As a result, we observed the little DeepDRiD dataset while simultaneously using the older Kaggle dataset in our investigations. We even achieved the top score using a different, smaller DeepDRiD dataset. With a higher F1 score of 0.87 and 0.84, the efficient net b5 and b6 models on the DeepDRiD and EYE-PACS datasets detected all types of DR. On the DeepDRiD dataset, however, we attained the greatest F1 score of 0.87. Our suggested research offers scores for smaller datasets like DeepDRiD in addition to S-O-T-A (state-of-the-art) outcomes for huge datasets like EYE-PACS. One disadvantage of datasets larger than 6GBs is that these CNN models cannot be trained on the available GPU. Due to the extensive parameters compared to other efficient net BNs that were used in our work, we were also unable to train EYE-PACS data using efficient net b7 due to the limited availability of GPU. The algorithms have a number of shortcomings that prevent them from classifying with high image resolution in datasets on Google-Colab, including extraordinarily high computational costs, the lack of use of complex preprocessing and data augmentation techniques, and the failure to add additional features to the models. These datasets may not fully represent the whole population because of biases against specific groups. This could limit how well the model works with various groups of the population. If the datasets span a sizable period of time, changes in imaging technology or clinical practices may result in temporal bias. It may be difficult to apply models created by old data to instances from more recent periods.

Other drawbacks include the deployment of automated categorization algorithms in healthcare contexts presents ethical and legal considerations. Clinicians may rely extensively on these models, thereby reducing their own diagnostic and decision-making abilities. Liability problems may occur if a misclassification results in negative patient consequences. In conclusion, while machine learning-based categorization of diabetic retinopathy offers promise, it is critical to recognize and address these possible disadvantages to ensure that the technology is utilized ethically and efficiently in clinical settings. The constraints can be overcome by validating the models’ performance via ensemble models, k-fold cross-validation, future modifications in model topologies, and other cutting-edge structural designs such as CoAtNet. We may also share data repositories to help Artificial Intelligence (AI) systems in the future by enabling collaborative learning and the development of more robust models. In essence, the dynamic nature of the medical industry creates the groundwork for AI to redefine diabetic retinopathy diagnosis. These advancements provide more accuracy, faster analysis, and personalized therapy, ensuring that diabetic patients receive earlier medications and better outcomes, ushering in a new era of precision medicine. Researchers are encouraged to explore the challenges associated with deploying Deep Learning (DL) models for Diabetic Retinopathy (DR) classification in real-world clinical settings. This exploration should encompass critical aspects such as data privacy concerns, regulatory compliance adherence, and seamless integration with electronic health records (EHRs). By focusing on these specific research directions, these emerging scholars have the potential to make substantial contributions to the field of diabetic retinopathy categorization. These contributions hold the promise of significantly enhancing the diagnosis, treatment, and overall management of this sight-threatening medical condition.

## Conclusion

Currently, a very limited investigation into separating all diabetic retinopathy groups with a higher F1 score has been completed. We used efficient net BNs models in our paper. It has been noted that grade 0 of DR occurs more frequently in other CNN models. In earlier research, we found that applying ResNet and VGG models, which were scaled only by their depth, suggested that they were unable to obtain the parameters of the images that resembled classes. Previously used efficient net models consistently scaled the depth, width, and resolution using the compound scaling technique More than one class of DR can be distinguished by the primary factor of efficient net BN models^13^. Future improvements will be needed, and these can be made by applying advanced preprocessing and data augmentation techniques, using images with the highest resolution possible, applying an ensemble of multiple CNN classification models, or using a model similar to this one but with additional features to achieve the best possible outcome.

## Data Availability

The data utilized in this investigation is openly accessible at https://github.com/deepdrdoc/Deep-Diabetic-Retinopathy-Image-Dataset-DeepDRiD-; https://www.kaggle.com/datasets/mariaherrerot/eyepacspreprocess.
